# Prenatal exposure to nitrofurantoin and risk of childhood leukaemia: a registry-based cohort study in four Nordic countries

**DOI:** 10.1093/ije/dyab219

**Published:** 2021-10-13

**Authors:** Sarah Hjorth, Anton Pottegård, Anne Broe, Caroline H Hemmingsen, Maarit K Leinonen, Marie Hargreave, Ulrika Nörby, Hedvig Nordeng

**Affiliations:** PharmacoEpidemiology and Drug Safety Research Group, Department of Pharmacy, and PharmaTox Strategic Initiative, Faculty of Mathematics and Natural Sciences, University of Oslo, Oslo, Norway; Clinical Pharmacology, Pharmacy and Environmental Medicine, Department of Public Health, University of Southern Denmark, Odense, Denmark; Clinical Pharmacology, Pharmacy and Environmental Medicine, Department of Public Health, University of Southern Denmark, Odense, Denmark; Department of Clinical Biochemistry and Pharmacology, Odense University Hospital, Odense, Denmark; Virus, Lifestyle and Genes, Danish Cancer Society Research Center, Copenhagen, Denmark; Data and Analytics, Information Services Department, Finnish Institute for Health and Welfare, Helsinki, Finland; Virus, Lifestyle and Genes, Danish Cancer Society Research Center, Copenhagen, Denmark; Health and Medical Care Administration, Region Stockholm, Stockholm, Sweden; PharmacoEpidemiology and Drug Safety Research Group, Department of Pharmacy, and PharmaTox Strategic Initiative, Faculty of Mathematics and Natural Sciences, University of Oslo, Oslo, Norway; Department of Child Health and Development, Norwegian Institute of Public Health, Oslo, Norway

**Keywords:** Leukaemia, nitrofurantoin, prenatal exposure, delayed effects

## Abstract

**Background:**

Studies have suggested increased risks of childhood leukaemia after prenatal exposure to antibiotics, particularly nitrofurantoin. However, these findings may be related to the underlying maternal infection. This multinational study aimed to investigate the association between prenatal nitrofurantoin exposure and childhood leukaemia while accounting for maternal infection.

**Methods:**

In a population-based cohort study of children born in Denmark, Finland, Norway or Sweden from 1997 to 2013, prenatal exposure to nitrofurantoin or pivmecillinam (active comparator) was ascertained from national Prescription Registries. Childhood leukaemia was identified by linkage to national Cancer Registries. Poisson regression was used to estimate incidence rate ratios (IRRs) and incidence rate differences (IRDs) with inverse probability of treatment weights applied to account for confounding.

**Results:**

We included 44 091 children prenatally exposed to nitrofurantoin and 247 306 children prenatally exposed to pivmecillinam. The children were followed for 9.3 years on average (standard deviation 4.1). There were 161 cases of childhood leukaemia. The weighted IRR for prenatal nitrofurantoin exposure when compared with pivmecillinam was 1.34 (95% confidence interval 0.88, 2.06), corresponding to an IRD of 15 per million person-years. Higher point estimates were seen for first- and third-trimester exposure. There was no evidence of a dose–response relationship.

**Conclusions:**

Prenatal exposure to nitrofurantoin was not substantially associated with childhood leukaemia, although a slightly elevated IRR with confidence intervals including the null was observed, corresponding to a small absolute risk. The lack of a dose–response relationship and a clear biological mechanism to explain the findings suggests against a causal association.

Key Messages

- Previous studies on prenatal exposure to antibiotics and childhood cancer have used unexposed comparators, which may introduce confounding from underlying maternal infection. Furthermore, findings based on a non-user comparator are of limited clinical value as urinary tract infections during pregnancy should always be treated.
- In a multinational cohort study, we investigated the association between prenatal exposure to nitrofurantoin and childhood leukaemia when compared with pivmecillinam, another antibiotic used for the same indication.
- There was no substantial association between prenatal exposure to nitrofurantoin and childhood leukaemia, albeit a slightly elevated incidence rate ratio with confidence intervals overlapping the null. The current body of evidence, including the lack of a clear biological mechanism of action, speaks against interpreting the association as causal.

## Background

Cancer is the second most common cause of death in children in affluent countries, with leukaemia as the most common type.^1^ Childhood cancer incidence in Scandinavia is 160 per million per year and leukaemia accounts for a third of cases.^1^ The aetiology of childhood leukaemia is largely unknown, but is thought to involve a combination of pre- and postnatal factors.^2^^,^^3^ A number of studies have investigated potential associations between prenatal exposure to antibiotics and risk of childhood leukaemia when compared with no exposure with heterogeneous findings.^4–8^ The only large study to investigate at the individual antibiotic substance level and specific types of childhood cancers found associations with leukaemia for exposure to some commonly used antibiotics, including nitrofurantoin [hazard ratio (HR) 1.56, 95% confidence interval (CI) 1.02, 2.37] when compared with unexposed children.^7^ However, maternal infection has also been proposed to affect the risk of childhood leukaemia.^2^ Therefore, it is important to also compare to children who were exposed to maternal infection but received another antibiotic treatment, i.e. use of an active comparator.^9^ This is particularly relevant for urinary tract infections in pregnancy, as treatment is always indicated, even in asymptomatic cases.^10^^,^^11^ Clinicians and pregnant women therefore need to choose between different antibiotic treatment regimens rather than between antibiotics or no treatment. Up to 10% of pregnancies are affected by bacteriuria, making it one of the most common pregnancy complications.^10^ The Nordic countries have similar clinical guidelines for the treatment of bacteriuria in pregnancy with pivmecillinam as the first-line treatment and nitrofurantoin as an equivalent or second-line treatment, depending on the country (Supplementary Material, available as Supplementary data at *IJE* online). In the above-mentioned study, based on Danish and Swedish data, pivmecillinam was not associated with childhood leukaemias (HR 1.11, 95% CI 0.83, 1.48).^7^ Therefore, pivmecillinam could be used as an active comparator to nitrofurantoin in a Nordic setting. The objective of the present study was to investigate the risk of childhood leukaemia after prenatal exposure to nitrofurantoin compared with pivmecillinam.

## Methods

The design was an active comparator cohort study using individual-level data from national registries in Denmark, Finland, Norway and Sweden. We estimated the incidence rate ratios (IRRs) and incidence rate differences (IRDs) for childhood leukaemia by comparing the incidence among children who were prenatally exposed to nitrofurantoin and children who were prenatally exposed to pivmecillinam.

### Data sources

We used data from Nordic Cancer Registries, Prescription Registries, Medical Birth Registries and Patient Registries. In addition, we used data from the Danish Civil Registration System and from the Cause of Death Registries in Finland and Sweden. In the Nordic countries, the registries cover all residents. Linkage between registries is made possible by unique personal identification numbers.

The Nordic Cancer Registries have recorded incident cases of cancer since 1942–1958, depending on the country, with a coverage of close to 100%.^12–15^ See Supplementary Figures S1 and S2 (available as Supplementary data at *IJE* online) for an overview of the periods covered by the registries in each country.

The Nordic Prescription Registries provide information on all prescriptions redeemed at pharmacies by patients in ambulatory care.^16^ Medications are categorized according to the Anatomical Therapeutic Chemical (ATC) Classification System.^17^ The Prescription Registries were established in 1993–2005.^16^

The Nordic Medical Birth Registries cover a wide range of information on ante- and perinatal factors, as well as some background information on mother, father and infant.^18^ Notification to the Medical Birth Registries has been mandatory for live births since 1967–1987, depending on the country.^18^

The Nordic Patient Registries record all diagnoses and procedures in government-owned hospitals and outpatient clinics. The Patient Registries have contained individual-level data since 1977–2008, depending on the country.^19–22^

The Nordic Cause of Death Registries record cause and date of death;^23^^,^^24^ the Civil Registries record dates of death and migration for all citizens.^25^

### Study sample

The initial study population was live-born singletons registered in the Nordic Medical Birth Registries. We included all children born between 1997 and 2013 in Denmark and Finland, and between 2007 and 2013 in Norway and Sweden, who were prenatally exposed to either nitrofurantoin or pivmecillinam. Exclusion criteria were prenatal exposure to both pivmecillinam and nitrofurantoin or inability to determine exposure to medications in pregnancy because of (i) missing maternal or child identification number or (ii) missing or unrealistic (>45 weeks) gestational age at birth and thus missing information about the start of pregnancy. Follow-up continued until the first cancer diagnosis, death (handled as a competing risk), emigration, the child’s twentieth birthday or 31 December 2017, whichever came first.

### Exposures

Exposure was maternal filling of one or more prescriptions of the medication of interest during pregnancy, as recorded in the Nordic Prescription Registries. Pivmecillinam was identified by ATC code J01CA08, nitrofurantoin by ATC code J01XE01.

Analysis was also performed for number of prescriptions filled in pregnancy (one fill of nitrofurantoin compared with one fill of pivmecillinam, and two or more fills of nitrofurantoin compared with two or more fills of pivmecillinam with the resulting two estimates compared to assess any dose–response relationship) and for the timing of prescription fills (first, second or third trimester, defined as days 0–89; 90–179 or birth, if the child was born during the second trimester; and 180 to birth). Day zero of pregnancy was defined as the first day of the last menstrual period, primarily estimated by ultrasound.

In the Nordic countries, antibiotics are only available on prescription, so the sensitivity of the exposure classification is expected to be high.

### Outcomes

The primary outcome was any leukaemia as recorded in the Nordic Cancer Registries [International Classification of Childhood Cancer, third edition (ICCC-3) site group 1, codes 011–015].^26^ As secondary outcomes, leukaemia was specified according to the most common types of childhood leukaemia, namely lymphoid leukaemia (ICCC-3 code 011) and acute myeloid leukaemia (ICCC-3 code 012).^26^

A validation study in the Finnish Cancer Registry showed 95.7% completeness of childhood-leukaemia registrations.^13^

### Covariates

Covariates were chosen a priori using subject knowledge and Directed Acyclic Graphs (Supplementary Figure S3, available as Supplementary data at *IJE* online).^27^ As the outcomes were expected to be very rare, a propensity-score-based approach to confounder adjustment was chosen. The propensity score was estimated using logistic regression.^28^ As recommended, the propensity score included both potential confounders and predictors of the outcome.^28^ The following covariates were included: calendar year at birth (numerical), child sex, maternal age (numerical), parity at start of pregnancy (0, 1, 2, 3, 4+), maternal history of cancer before pregnancy (yes/no), maternal smoking status during first trimester (yes/no) and prescription fills for immunosuppressants (ATC code L04), systemic corticosteroids (ATC codes H02A and H02B) or systemic antibiotics (ATC code J01, other than pivmecillinam and nitrofurantoin) in the year before (in Finland 3 months before) start of pregnancy (yes/no, used as a proxy for susceptibility to infections). Information on covariates was obtained from the Medical Birth Registries, the Prescription Registries and the Cancer Registries. Smoking in pregnancy is highly correlated with socio-economic position in the Nordic countries^29–31^ and was considered as a proxy for socio-economic position.

### Statistical analysis

Baseline characteristics and antibiotic utilization (number of fills in pregnancy, trimester of fills and number of fills for other antibiotics in pregnancy) were compared between pivmecillinam-exposed and nitrofurantoin-exposed children. Analyses were conducted separately for each country. To allow for different lengths of follow-up, we used Poisson regression to obtain IRRs and IRDs. Crude estimates with 95% CIs were obtained using generalized linear models with a log link. To account for confounding, propensity scores were estimated using logistic regression and used as inverse probability of treatment weights (IPTW). IPTW is recommended when using active comparators^32^ and will (under the assumption of no residual confounding) answer the question: What would the incidence rate have been if everyone had been treated with nitrofurantoin as opposed to if everyone had been treated with pivmecillinam? The non-overlapping regions of the propensity score were trimmed (59 children excluded), with the baseline characteristics of the mothers to the excluded children found to be similar to the remaining cohort (data not shown for reasons of confidentiality due to the small numbers). The balance of the weights was checked using standardized mean differences.^33^ Covariates were considered balanced if the standardized mean differences were <0.1.^33^ Generalized linear models with robust standard errors were used in the weighted data set to obtain weighted estimates with 95% CIs. Robust standard errors were chosen to account for weighting and clustering, as more than one child of the same mother could be included in the cohort.

Missing data on covariates (smoking and parity) were seen for 6.9% of the study sample. Under the assumption that data were missing at random, missing data were imputed using multiple imputation by chained equations^34^ with 50 data sets created. As done in previous studies using Poisson regression, the imputation model included exposure, outcome, all covariates and the cumulative Nelson Aalen hazard function for leukaemia.^35^

Fixed-effects meta-analysis was used to pool the results from Danish, Finnish, Norwegian and Swedish data, assuming a common treatment effect across the Nordic countries.^36^ In a Nordic context, fixed-effects meta-analysis has been shown to yield results similar to those obtained from pooling individual data.^37^ Heterogeneity was examined using *I*^2^. For analyses in which there were zero exposed or unexposed cases in one or more countries, results were combined using multilevel mixed-effects Poisson regression with random-effect terms for the variance components.^38^ This method has been shown to yield results with less bias than standard meta-analysis techniques in the meta-analyses of incidence rate data with zero counts.^38^

### Supplementary analyses

We performed several pre-planned supplementary analyses to assess the robustness of our findings.

First, to estimate the impact of potential unmeasured confounding, e.g. by severity of infection, we calculated the e-value. The e-value was calculated to examine how strong any unmeasured confounding should be to explain the observed effect to the extent that it reduces the observed point estimate to the null.^39^

Second, to compare with the results from the imputed data set, we did a complete case analysis.

Third, we started follow-up at 1 year of age, as infant leukaemia may have a different aetiology from later-onset childhood leukaemia.^2^

Fourth, to strengthen the comparability between nitrofurantoin-exposed and pivmecillinam-exposed children, we restricted the sample to those who were unexposed to systemic antibiotics *in utero* other than pivmecillinam or nitrofurantoin.

Fifth, we restricted the sample to women who had had contact with the healthcare system (diagnosis or prescription fill) before pregnancy. This was expected to ensure that women were present in the country throughout the pregnancy. However, this analysis was also expected to restrict the sample to women with more co-morbidities than the general population.

### *Post hoc* analysis

In a *post hoc* analysis to estimate the extent of the confounding by indication, we compared children prenatally exposed to nitrofurantoin to children who were unexposed to antibiotics in pregnancy. The same variables were included in the propensity scores as in the main analysis but standardized mortality ratio weights were used, as recommended for population comparators.^32^

## Results

**Figure 1:**
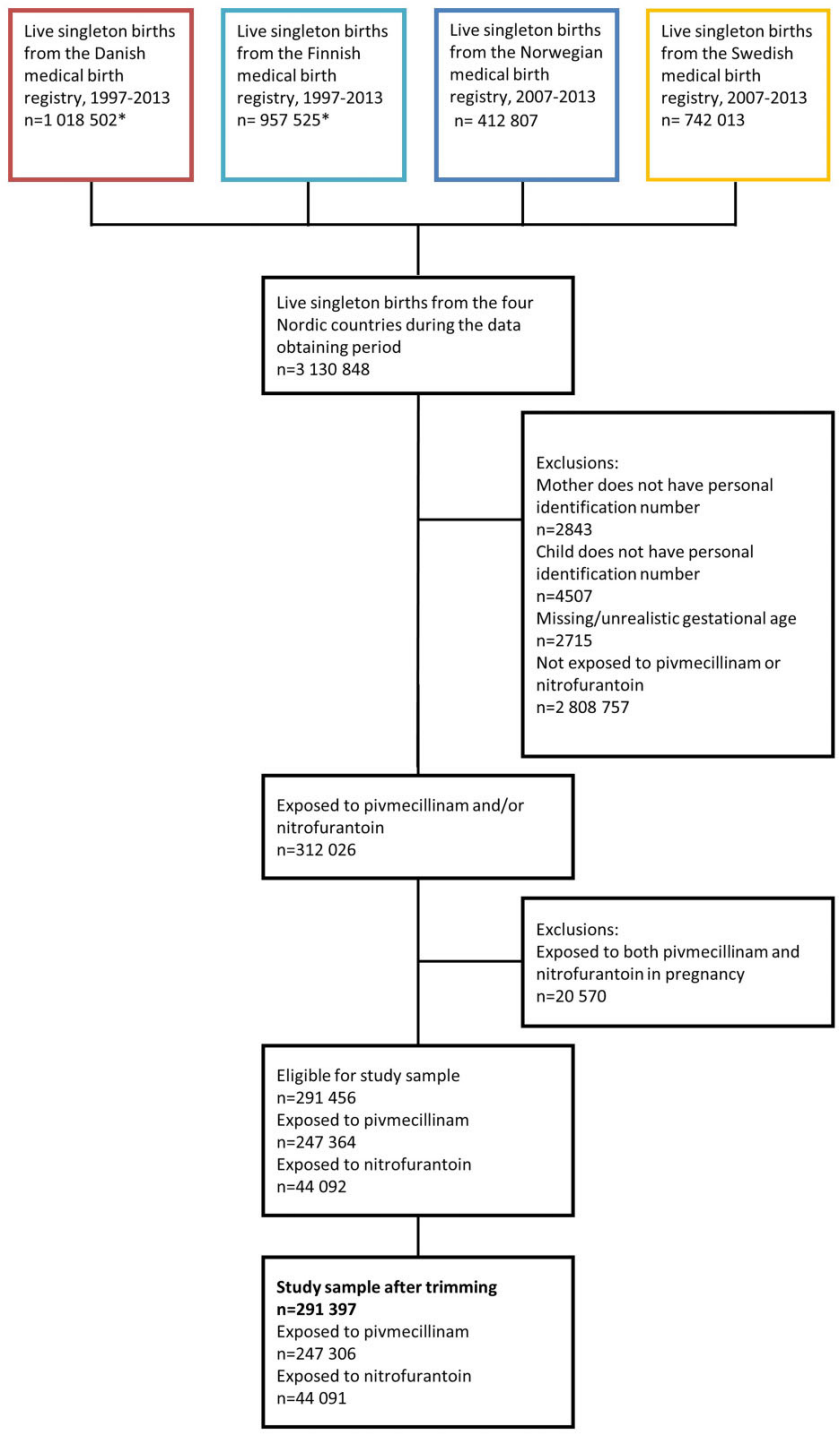
Flowchart of the study population. *Individuals with missing identification number or gestational age were excluded by registry holders, so we do not have information on how many individuals were excluded for these reasons.

Among 3 135 376 live-born children, we identified 312 026 (10.0%) children prenatally exposed to nitrofurantoin and/or pivmecillinam (Figure 1). Of these, 20 570 children, corresponding to 7.7% of pivmecillinam-exposed and 31.8% of nitrofurantoin-exposed, were excluded due to exposure to both medications. We included 44 091 (1.4%) prenatally nitrofurantoin-exposed and 247 306 (7.9%) pivmecillinam-exposed children. Pivmecillinam-exposed children were more often exposed to maternal smoking in pregnancy and to more than one treatment with the antibiotic medication of interest (Table 1). The prevalence of prenatal exposure to other systemic antibiotics was similar between nitrofurantoin-exposed and pivmecillinam-exposed children. All covariates included in the IPTW had a standardized mean difference of <0.1 after weighting. An exception was the model for Finland, in which the birth year was not balanced and was hence added to the outcome model. In all countries, the weights had a mean of 1.00. The highest weight in any country was 3.64.

**Table 1:** Characteristics of included pregnancies exposed to nitrofurantoin or pivmecillinam according to the Nordic Prescription Registries

	Exposed to nitrofurantoin (*n* = 44 091)	Exposed to pivmecillinam (*n* = 247 306)
Maternal age (mean [sd])	29.6 (5.5)	29.0 (5.4)
Maternal nulliparity [*n* (%)]	20 607 (46.7)	119 069 (48.1)
Maternal history of cancer before pregnancy [*n* (%)]	276 (0.6)	1356 (0.5)
Maternal smoking in early pregnancy [*n* (%)]	5279 (12.0)	40 923 (16.5)
Child country of birth [*n* (%)]		
Denmark	8794 (19.9)	118 303 (47.8)
Finland	1210 (2.7)	44 422 (18.0)
Norway	6908 (15.7)	50 899 (20.6)
Sweden	27 179 (61.6)	33 682 (13.6)
Child male sex [*n* (%)]	22 611 (51.3)	127 091 (51.4)
Number of prescription fills in the year before pregnancy [*n* (%)]		
Nitrofurantoin		
0	41 243 (93.5)	242 714 (98.1)
1	2229 (5.1)	3887 (1.6)
2+	619 (1.4)	705 (0.3)
Pivmecillinam		
0	39 591 (89.8)	219 623 (88.8)
1	3664 (8.3)	21 045 (8.5)
2+	836 (1.9)	6638 (2.7)
Number of prescription fills for study medication in pregnancy [*n* (%)]		
1	38 448 (87.2)	199 598 (80.7)
2+	5643 (12.8)	47 708 (19.3)
Trimester of exposure		
First	11 688 (26.5)	75 160 (30.4)
Second	15 657 (35.5)	88 231 (35.7)
Third	20 460 (46.4)	116 817 (47.2)
Number of prescription fills for other antibiotics in pregnancy [*n* (%)]		
0	29 554 (67.0)	166 635 (67.4)
1	9358 (21.2)	53 404 (21.6)
2+	5179 (11.7)	27 267 (11.0)

The included children were followed for 984 784 867 person-years in total, with each child followed for 9.3 years on average (standard deviation 4.1). During follow-up, there were 161 cases of leukaemia, 134 of which were lymphoid leukaemia. Compared with pivmecillinam, prenatal nitrofurantoin exposure was associated with a slightly elevated IRR for leukaemia (fixed-effects IRR 1.34, 95% CI 0.88, 2.06, *I*^2^ = 0), albeit with wide confidence intervals that included the null (Table 2). This corresponds to an IRD of 15 per million person-years. For almost all analyses, there were zero exposed or unexposed cases in at least one country. Therefore, remaining analyses were combined using mixed-effects Poisson models. The IRR comparing children with one prenatal nitrofurantoin exposure to children with one prenatal pivmecillinam exposure was 1.25 (95% CI 0.77, 2.04). The statistical precision was limited, but we found no evidence of a larger increase in leukaemia incidence after two or more prenatal nitrofurantoin exposures when compared with two or more pivmecillinam exposures (IRR 1.57, 95% CI 0.54, 4.55). Trimester-specific analyses pointed to increased incidences of leukaemia after first- and third-trimester nitrofurantoin exposure, but not after second-trimester exposure.

**Table 2:** Incidence rate ratios and differences for leukaemia comparing children prenatally exposed to nitrofurantoin and pivmecillinam

		Any leukaemia					Lymphoid leukaemia					AML
	*n*	Cases	IRR (95% CI)	wIRR^b^ (95% CI)	IRD pr 100 000 person-years (95% CI)	wIRD^b^ pr 100 000 person-years (95% CI)	Cases	IRR (95% CI)	wIRR^b^ (95% CI)	IRD pr 100 000 person-years (95% CI)	wIRD^b^ pr 100 000 person-years (95% CI)	Cases
Pivmecillinam	247 306	129	Reference				108	Reference				13
Nitrofurantoin	44 091	32	1.36 (0.90, 2.06)^a^	1.34 (0.88,2.06)^a^	1.90 (–1.42, 5.23)^a^	1.49 (–1.92, 4.90)^a^	26	1.42 (0.92, 2.17)	1.34 (0.83, 2.17)	1.95 (–0.89, 4.79	1.60 (–1.46, 4.66)	<5
Two or more prescription fills												
Pivmecillinam	47 708	23	Reference				17	Reference				<5
Nitrofurantoin	5643	6	2.15 (0.88, 5.28)	1.57 (0.54, 4.55)	6.24 (–4.24, 16.74)	3.09 (–5.92, 12.10)	<5	–	–	–	–	<5
Trimester of exposure												
First trimester												
Pivmecillinam	75 160	26	Reference				24	Reference				<5
Nitrofurantoin	11 688	7	1.83 (0.79, 4.21)	1.92 (0.84, 4.41)	3.11 (–2.61, 8.82)	3.53 (–2.60, 9.65)	5	1.42 (0.54, 3.71)	1.48 (0.57, 3.87)	1.44 (–3.28, 6.15)	1.71 (–3.35, 6.76)	<5
Second trimester												
Pivmecillinam	88 231	48	Reference				44	Reference				0
Nitrofurantoin	15 657	6	0.74 (0.32, 1.72)	0.53 (0.19, 1.47)	–1.58 (–5.31, 2.16)	–2.77 (–5.97, 0.43)	5	0.67 (0.27, 1.69)	0.58 (0.21, 1.61)	–1.81 (–5.20, 1.58)	–2.28 (–5.49, 0.93)	<5
Third trimester												
Pivmecillinam	116 817	67	Reference				51	Reference				11
Nitrofurantoin	20 460	21	1.73 (1.03, 2.92)	1.73 (1.00, 2.98)	4.81 (–1.13, 10.76)	4.77 (–1.37, 10.90)	16	1.63 (0.89, 3.00)	1.66 (0.88, 3.11)	3.31 (–1.90, 8.52)	3.44 (–2.01, 8.88)	<5

a Results from fixed-effects meta-analysis, *I*^2^ = 0.0%. Findings from mixed-effects Poisson models were comparable: IRR 1.44 (0.96, 2.17), wIRR 1.40 (0.91, 2.15).

b Inverse probability of treatment weights including calendar year at birth, maternal age, parity, maternal history of cancer before pregnancy, prescription fills for immunosuppressants, systemic corticosteroids and systemic antibiotics before start of pregnancy, maternal smoking status during first trimester and child sex. In Finland, birth year was not balanced after weighting and hence was added to the outcome model.

AML, acute myeloid leukaemia; IRD, incidence rate difference; IRR, incidence rate ratio; wIRD, weighted incidence rate difference; wIRR, weighted incidence rate ratio.

In the secondary analyses on lymphoid leukaemia, nitrofurantoin exposure was associated with an IRR of 1.34 (95% CI 0.83, 2.17). Analyses on acute myeloid leukaemia were not feasible due to the small number of cases.

### Supplementary analyses

In general, the statistical precision was low in the supplementary analyses, but results corresponded with results from the main analysis (Supplementary Tables S1–S4, available as Supplementary data at *IJE* online).

For the association between any prenatal nitrofurantoin exposure and childhood leukaemia, the e-value was 2.02, meaning that any unmeasured confounder would have to have an association of 2.02 with both the exposure and the outcome to fully explain the IRR.

### Post hoc *analysis*

The *post hoc* analysis compared the 44 091 children prenatally exposed to nitrofurantoin to 2 254 684 unexposed children. Findings were similar to the results from the active comparator design (IRR 1.23, 95% CI 0.87, 1.76, IRD 14 per million person-years) (Supplementary Table S5, available as Supplementary data at *IJE* online).

## Discussion

In this active comparator study of 291 397 children from four Nordic countries, we found no substantial association between prenatal exposure to nitrofurantoin and childhood leukaemia, although we observed a slightly elevated IRR with wide confidence intervals overlapping the null. There was no evidence of a dose–response relationship. The results were stable in supplementary analyses.

Our findings are in accordance with the previous study on Danish and Swedish data that used population comparators,^7^ although point estimates in the present study are lower. Our study sample partially overlaps with the sample from that study by three of the included years from Sweden and 11 of the included years from Denmark.

We could only estimate associations between trimester-specific exposure and childhood leukaemia with imprecision, but first- and third-trimester exposures were associated with the largest estimates of association. Previous studies did not investigate nitrofurantoin exposure by trimester, but one Canadian study has investigated exposure to any antibiotic by trimester.^8^ That study indicated an increased risk of childhood acute lymphoid leukaemia after first-trimester exposure to antibiotics (HR 1.5, 95% CI 0.9, 2.5), but not after second- or third-trimester exposure (both HR 0.8).^23^

A biological mechanism that could explain the findings is lacking, as there has not been evidence to suggest that even high doses of nitrofurantoin can cause leukaemia in animals.^40^

Strengths of this study include the population-based design with information on all exposed pregnancies across four Nordic countries and follow-up in nationwide registries of high completeness.^13^ Another strength is the use of an active comparator design to account for confounding by maternal infection and aid clinical decision-making.

Whereas maternal infection in pregnancy has been proposed as a risk factor for childhood leukaemia,^2^ few studies have investigated this for maternal urinary tract infections.^41^ A systematic review from 2020 identified two studies on prenatal exposure to maternal urinary tract infection and childhood lymphoid leukaemia^41^ in which one found an odds ratio of 0.7 (95% CI 0.4, 1.2) and the other an odds ratio of 1.9 (95% CI 1.0, 3.9). The study that found a point estimate of >1 used information from medical records, whereas the other study relied on retrospective maternal report in which recall bias could occur. Therefore, an association between maternal urinary tract infection and childhood leukaemia cannot be ruled out. The biological mechanism of action is speculative, but maternal urinary tract infections can affect the fetal environment, as they are implicated in preterm births and low birthweight.^10^ Findings from the *post hoc* analysis suggested against important confounding by maternal urinary tract infection. However, confounding by other unmeasured factors cannot be ruled out, especially given that pivmecillinam does not appear to be a perfect active comparator to nitrofurantoin. The prevalence of exposure to nitrofurantoin or pivmecillinam across the four countries suggests that the two medications are only used as equivalents in Sweden. It is nonetheless reassuring that the meta-analysis of the results did not suggest important heterogeneity between the estimates from individual countries. Calculation of the e-value showed that a confounder with an association of 2.02 with both the exposure and the outcome could fully explain the IRR in the present study.

Another limitation is the potential misclassification of the exposures, as non-adherence to prescribed antibiotics in pregnancy has been reported.^42^ A Danish study found a sensitivity of 93% and a specificity of 88% for antibiotic-prescription fills when compared with self-report in prospective biweekly questionnaires.^42^ It is unknown whether such non-adherence differs by antibiotic substance. If so, the direction of the resulting bias would be unpredictable, as there would be three exposure groups in our study: pivmecillinam-exposed, nitrofurantoin-exposed and children exposed to untreated maternal infection. However, we had access to information on dispensed antibiotics, which is a better approximation of medication use than prescription records.^43^

Although the present study is based on data from four countries, the low number of exposed cases, especially in the dose–response analysis, is a limitation. Even in the main analysis, the confidence interval included a doubling of the incidence of leukaemia among nitrofurantoin-exposed children, highlighting a need for additional studies to assess this association. Nevertheless, even a doubling of the incidence rate translates into a very small increase on an absolute scale. As such, the present study does allow us to rule out a major public health impact on childhood-leukaemia incidence from nitrofurantoin treatment during pregnancy.

We did not have information about the ethnicity of the children included in the study sample. With a majority of Caucasian ethnicity in the Nordic countries and differences in disease susceptibility and drug responses between different ethnic groups, the findings from the present study may not be generalizable to populations with a different genetic composition.

In conclusion, we found no substantial association between childhood leukaemia and prenatal exposure to nitrofurantoin, albeit a slightly elevated IRR with confidence intervals including the null and corresponding to a small absolute risk. There was no dose–response relationship and a biologically plausible mechanism of action is lacking. Jointly, this suggests against a causal interpretation of the elevated IRR. Further studies are needed to provide additional data, as the statistical precision of the present study was limited.

## Supplementary data

Supplementary data are available at *IJE* online.

## Ethics approval

The study was approved by the Regional Committee for Medical Research Ethics in South-Eastern Norway (approval number: 2018/142/REK Sør-Øst) and by the Swedish Ethical Review Authority [approval number: 2018/2604–31/1 2019–00268 (2019–02311)]. In Denmark and Finland, registry-based studies are exempt from ethical review. The study was approved by local data-protection officers (approval number, Denmark: 2019-DCRC-0096; approval numbers, Finland: THL/2297/5.05.00/2018, Kela 120/522/2019, TK-53–1405-19; approval number, Norway: 233835). Data were handled in accordance with the research approvals and the applied legal norms including European Union General Data Protection Regulation (2016/79).

## Funding

The work was supported by the Nordic Cancer Union [grant number R275-A15824] and the European Research Council Starting Grant ‘DrugsInPregnancy’ [grant number 639377 to S.H. and H.N.].

## Data availability

The data underlying this article were provided by the registry holders by permission. Data will be shared on request to the corresponding author with the permission of the registry holders and the required ethical approvals.

## Supplementary Material

dyab219_Supplementary_Data
