# Correction to: Prenatal exposure to nitrofurantoin and risk of childhood leukaemia: a registry-based cohort study in four Nordic countries

**DOI:** 10.1093/ije/dyab271

**Published:** 2022-01-12

**Authors:** Sarah Hjorth, Anton Pottegård, Anne Broe, Caroline H Hemmingsen, Maarit K Leinonen, Marie Hargreave, Ulrika Nörby, Hedvig Nordeng

Published online: 13 October 2021, *International Journal of Epidemiology*, dyab219, https://doi.org/10.1093/ije/dyab219

The number of live-born singletons in Norway was presented incorrectly in Figure 1. The correct number is 412,807. This means that the total number of live-born singletons in the study period was 3,130,848 and the number of children unexposed to pivmecillinam and nitrofurantoin was 2,808,757. The error did not affect the included study sample, or any other results.

