# Supplementary material for: Prenatal exposure to nitrofurantoin and risk of childhood leukaemia: a registry-based cohort study in four Nordic countries
Source: Int J Epidemiol. 2021 Oct 13;51(3):778–88. doi: 10.1093/ije/dyab219 (PMC9189954; doi:10.1093/ije/dyab219)
Supplement: dyab219_Supplementary_Data [file dyab219_supplementary_data.docx]

**Supplementary information to**

**Prenatal exposure to nitrofurantoin and risk of childhood leukaemia – a registry-based cohort study in four Nordic countries**

Sarah Hjorth, Anton Pottegård, Anne Broe, Caroline H. Hemmingsen, Maarit K. Leinonen, Marie Hargreave, Ulrika Nörby, Hedvig Nordeng

**Contents:**

Supplementary information: Guidelines for the treatment of urinary tract infections in pregnancy in the Nordic countries, periods of data inclusion for the different national registries

Supplementary Table S1: Complete case analysis.

Supplementary Table S2: Analysis excluding cases of infant leukaemia.

Supplementary Table S3: Analysis excluding children prenatally exposed to other antibiotics.

Supplementary Table S4: Analysis restricted to children who are followed for the entire pregnancy.

Supplementary Table S5: *Post hoc* analysis comparing children prenatally exposed and unexposed to nitrofurantoin.

Supplementary Figure S1: Data included in the study

Supplementary Figure S2: Study design diagram

Supplementary Figure S3: Directed acyclic graph

**Guidelines for the treatment of urinary tract infections in pregnancy:**

| Denmark (1)  Asymptomatic bacteriuria or cystitis:   1. Pivmecillinam 2. Nitrofurantoin (do not use in last weeks of pregnancy)   Recurrent cystitis:   1. Pivmecillinam or nitrofurantoin |
| --- |
| Norway (2)  Asymptomatic bacteriuria or cystitis:   1. Pivmecillinam 2. Nitrofurantoin   Recurrent cystitis:   1. Pivmecillinam 2. Nitrofurantoin |
| Sweden (3)  Asymptomatic bacteriuria or cystitis:   1. Pivmecillinam or nitrofurantoin 2. Cefadroxil   Recurrent cystitis:   1. Nitrofurantoin or cefadroxil |
| Finland (4)  Asymptomatic bacteriuria or cystitis:   1. Pivmecillinam, nitrofurantoin (do not use in pregnancy weeks 38-42), amoxicillin, or first generation cephalosporins.   Recurrent cystitis:   1. Nitrofurantoin or methenamine hippurate |

**Sources:**

1. Böcher S, Holm A, Christiansen M, Bojer D, Kjeldsen HC. Urinvejsinfektion og asymptomatisk bakteriuri hos gravide. In: Lægehåndbogen [Internet]. København: DADL; 2019 [cited 2021 Apr 8]. Available from: <https://www.sundhed.dk/sundhedsfaglig/laegehaandbogen/obstetrik/tilstande-og-sygdomme/svangerskabsgener/urinvejsinfektion-og-asymptomatisk-bakteriuri-hos-gravide/>
2. Bærheim A, Grude N. Urinveisinfeksjon [Internet]. Antibiotikabruk i primærhelsetjenesten. 2019 [cited 2021 Apr 9]. Available from: <http://www.antibiotikaiallmennpraksis.no/index.php?action=showtopic&topic=vXmA4Spa>
3. Läkemedelsbehandling av urinvägsinfektioner i öppenvård – behandlingsrekommendation. Information från Läkemedelsverket. 2017; 28(5):21-36.
4. Wuorela M, Arikoski P, Kouri T, Laato M, Rannikko J, Sipilä R, et al. Virtsatieinfektiot [Internet]. Käypä Hoito. 2020 [cited 2021 Apr 29]. Available from: <https://www.kaypahoito.fi/hoi10050>

**Periods of data inclusion for the different national registries:**

| **Country** | **Years of data inclusion** | |
| --- | --- | --- |
| *Cancer registries* | | |
| Denmark | | 1943-2017 |
| Finland | | 1953-2017 |
| Norway | | 1953-2017 |
| Sweden | | 1958-2017 |
| *Prescription registries* | | |
| Denmark | | 1995-2013 |
| Finland | | 1996-2013 |
| Norway | | 2004-2013 |
| Sweden | | 2005-2013 |
| *Medical birth registries* | | |
| Denmark | | 1997-2013 |
| Finland | | 1997-2013 |
| Norway | | 2007-2013 |
| Sweden | | 2007-2013 |
| *Patient registries* | | |
| Denmark | | 1995-2013 |
| Finland | | 1996-2013 |
| Norway | | 2008-2013 |
| Sweden | | 1997-2013 |
| *Civil Registration System^a^* | | |
| Denmark | | 1996-2017 |
| *Cause of death registries^a^* | | |
| Finland | | 1997-2017 |
| Sweden | | 2007-2017 |

^a^In Denmark, data on death and migration were obtained from the Civil Registration System, in Finland and Sweden, data on death were obtained from the cause of death registries. In Norway, data on death and migration were obtained from the Medical Birth Registry.

**Supplementary Table S1: Incidence rate ratios and differences for leukaemia comparing children prenatally exposed to nitrofurantoin and pivmecillinam. Complete case analysis.^a^**

|  |  | Any leukaemia | | | | |  | Lymphoid leukaemia | | | | AML |
| --- | --- | --- | --- | --- | --- | --- | --- | --- | --- | --- | --- | --- |
|  | n | cases | IRR (95% CI) | wIRR^b^ (95% CI) | IRD pr 100,000 person-years (95% CI) | wIRD^b^ pr 100,000 person-years (95% CI) | cases | IRR (95% CI) | wIRR^b^ (95% CI) | IRD pr 100,000 person-years (95% CI) | wIRD^b^ pr 100,000 person-years (95% CI) | cases |
| Pivmecillinam | 230 627 | 118 | reference |  |  |  | 99 | reference |  |  |  | 11 |
| Nitrofurantoin | 41,066 | 30 | 1.50 (1.01;2.24) | 1.44 (0.91;2.27) | 2.78 (-0.55;6.11) | 2.47 (-1.21;6.15) | 24 | 1.43 (0.92;2.24) | 1.41 (0.89;2.24) | 2.01 (-0.96;4.97) | 1.93 (-1.12;4.97) | <5 |
| 2 or more prescription fills | | | | | | | | | | | | |
| Pivmecillinam | 44 482 | 22 | reference |  |  |  | 16 | reference |  |  |  | <5 |
| Nitrofurantoin | 5290 | 6 | 2.24 (0.91;5.53) | 1.90 (0.64;5.57) | 6.96 (-4.38;18.30) | 4.31 (-5.52;14.13) | <5 | - | - | - | - | <5 |
| Trimester of exposure | | | | | | | | | | | | |
| 1st trimester | | | | | | | | | | | | |
| Pivmecillinam | 70 109 | 24 | reference |  |  |  | 22 | reference |  |  |  | <5 |
| Nitrofurantoin | 10 907 | 6 | 1.71 (0.70;4.17) | 1.48 (0.57;3.86) | 2.63 (-3.06;8.33) | 1.83 (-3.61;7.27) | <5 | - | - | - | - | <5 |
| 2nd trimester | | | | | | | | | | | | |
| Pivmecillinam | 82 377 | 43 | reference |  |  |  | 39 | reference |  |  |  | 0 |
| Nitrofurantoin | 14 632 | 5 | 0.69 (0.27;1.74) | 0.59 (0.21;1.65) | -1.80 (-3.11;5.72) | -2.33 (-5.79;1.13) | <5 | - | - | - | - | <5 |
| 3rd trimester | | | | | | | | | | | | |
| Pivmecillinam | 108 904 | 62 | reference |  |  |  | 48 | reference |  |  |  | 9 |
| Nitrofurantoin | 19 041 | 21 | 1.86 (1.09;3.18) | 1.76 (0.99;3.13) | 5.80 (-0.90;12.51) | 5.04 (-1.65;11.73) | 16 | 1.72 (0.93;3.20) | 1.74 (0.92;3.31) | 3.93 (-1.85;9.72) | 4.03 (-2.00;10.05) | <5 |

^a^Results from mixed effects Poisson models.

^b^Inverse probability of treatment weights including calendar year at birth, maternal age, parity, maternal history of cancer before pregnancy, prescription fills for immunosuppressants, systemic corticosteroids, and systemic antibiotics before start of pregnancy, maternal smoking status during first trimester, and child sex. In Finland, birth year was not balanced after weighting and added to the outcome model.

IRD: Incidence rate difference; IRR: Incidence rate ratio; wIRD: weighted incidence rate difference; wIRR: weighted incidence rate ratio.

**Supplementary Table S2: Incidence rate ratios and differences for leukaemia comparing children prenatally exposed to nitrofurantoin and pivmecillinam. Infant leukaemia excluded.^a^**

|  |  | Any leukaemia | | | | |  | Lymphoid leukaemia | | | | AML |
| --- | --- | --- | --- | --- | --- | --- | --- | --- | --- | --- | --- | --- |
|  | n | cases | IRR (95% CI) | wIRR^b^ (95% CI) | IRD pr 100,000 person-years (95% CI) | wIRD^b^ pr 100,000 person-years (95% CI) | cases | IRR (95% CI) | wIRR^b^ (95% CI) | IRD pr 100,000 person-years (95% CI) | wIRD^b^ pr 100,000 person-years (95% CI) | cases |
| Pivmecillinam | 245 555 | 122 | reference |  |  |  | 104 | reference |  |  |  | 10 |
| Nitrofurantoin | 43 882 | 28 | 1.34 (0.87;2.08) | 1.30 (0.82;2.05) | 2.03 (-1.44;5.50) | 1.81 (-1.75;5.37) | 24 | 1.36 (0.88;2.13) | 1.31 (0.82;2.09) | 1.84 (-1.22;4.90) | 1.56 (-1.54;4.66) | <5 |
| *2 or more prescription fills* | | | | | | | | | | | | |
| Pivmecillinam | 47 380 | 23 | reference |  |  |  | 17 | reference |  |  |  | <5 |
| Nitrofurantoin | 5612 | <5 | - | - | - | - | <5 | - | - | - | - | <5 |
| *Trimester of exposure* | | | | | | | | | | | | |
| *1st trimester* | | | | | | | | | | | | |
| Pivmecillinam | 74 648 | 25 | reference |  |  |  | 23 | reference |  |  |  | <5 |
| Nitrofurantoin | 11 628 | 6 | 1.64 (0.67;4.00) | 1.74 (0.72;4.23) | 2.59 (-3.32;8.50) | 3.06 (-3.33;9.45) | 5 | 1.48 (0.56;3.92) | 1.57 (0.59;4.14) | 1.79 (-3.54;7.11) | 2.16 (-3.63;7.95) | <5 |
| *2nd trimester* | | | | | | | | | | | | |
| Pivmecillinam | 87 584 | 47 | reference |  |  |  | 44 | reference |  |  |  | 0 |
| Nitrofurantoin | 15 587 | <5 | - | - | - | - | <5 | - | - | - | - | 0 |
| *3rd trimester* | | | | | | | | | | | | |
| Pivmecillinam | 115 996 | 62 | reference |  |  |  | 48 | reference |  |  |  | 9 |
| Nitrofurantoin | 20 362 | 18 | 1.63 (0.93;2.85) | 1.69 (0.95;3.01) | 4.22 (-1.90;10.34) | 4.67 (-1.87;11.21) | 15 | 1.68 (0.90;3.12) | 1.64 (0.86;3.13) | 3.68 (-1.99;9.34) | 3.55 (-2.36;9.45) | <5 |

^a^Results from mixed effects Poisson models.

^b^Inverse probability of treatment weights including calendar year at birth, maternal age, parity, maternal history of cancer before pregnancy, prescription fills for immunosuppressants, systemic corticosteroids, and systemic antibiotics before start of pregnancy, maternal smoking status during first trimester, and child sex. In Finland, birth year was not balanced after weighting and added to the outcome model.

IRD: Incidence rate difference; IRR: Incidence rate ratio; wIRD: weighted incidence rate difference; wIRR: weighted incidence rate ratio.

**Supplementary Table S3: Incidence rate ratios and differences for leukaemia comparing children prenatally exposed to nitrofurantoin and pivmecillinam. Children prenatally exposed to other antibiotics excluded.^a^**

|  |  | Any leukaemia | | | | |  | Lymphoid leukaemia | | | | AML |
| --- | --- | --- | --- | --- | --- | --- | --- | --- | --- | --- | --- | --- |
|  | n | cases | IRR (95% CI) | wIRR^b^ (95% CI) | IRD pr 100,000 person-years (95% CI) | wIRD^b^ pr 100,000 person-years (95% CI) | cases | IRR (95% CI) | wIRR^b^ (95% CI) | IRD pr 100,000 person-years (95% CI) | wIRD^b^ pr 100,000 person-years (95% CI) | cases |
| Pivmecillinam | 166 635 | 77 | reference |  |  |  | 66 | reference |  |  |  | 7 |
| Nitrofurantoin | 29 554 | 21 | 1.55 (0.92;2.61) | 1.57 (0.92;2.67) | 2.90 (-1.34;7.14) | 2.95 (-1.40;7.31) | 19 | 1.63 (0.94;2.82) | 1.63 (0.92;2.86) | 2.85 (-1.21;6.90) | 2.82 (-1.33;6.97) | <5 |
| *2 or more prescription fills* | | | | | | | | | | | | |
| Pivmecillinam | 28 084 | 11 | reference |  |  |  | 7 | reference |  |  |  | 0 |
| Nitrofurantoin | 3097 | <5 | - | - | - | - | <5 | - | - | - | - | <5 |
| *Trimester of exposure* | | | | | | | | | | | | |
| *1st trimester* | | | | | | | | | | | | |
| Pivmecillinam | 50 440 | 12 | reference |  |  |  | 11 | reference |  |  |  | 0 |
| Nitrofurantoin | 8163 | <5 | - | - | - | - | <5 | - | - | - | - | <5 |
| *2nd trimester* | | | | | | | | | | | | |
| Pivmecillinam | 57 652 | 29 | reference |  |  |  | 26 | reference |  |  |  | 0 |
| Nitrofurantoin | 10 361 | <5 | - | - | - | - | <5 | - | - | - | - | <5 |
| *3rd trimester* | | | | | | | | | | | | |
| Pivmecillinam | 77 173 | 41 | reference |  |  |  | 33 | reference |  |  |  | 6 |
| Nitrofurantoin | 13 031 | 15 | 2.08 (1.08;3.98) | 2.03 (1.04;3.98) | 6.79 (-1.74;15.32) | 6.48 (-2.11;15.07) | 13 | 2.15 (1.05;4.39) | 2.04 (0.98;4.29) | 5.99 (-2.01;13.98) | 5.44 (-2.44;13.33) | <5 |

^a^Results from mixed effects Poisson models.

^b^Inverse probability of treatment weights including calendar year at birth, maternal age, parity, maternal history of cancer before pregnancy, prescription fills for immunosuppressants, systemic corticosteroids, and systemic antibiotics before start of pregnancy, maternal smoking status during first trimester, and child sex. In Finland, birth year was not balanced after weighting and added to the outcome model.

IRD: Incidence rate difference; IRR: Incidence rate ratio; wIRD: weighted incidence rate difference; wIRR: weighted incidence rate ratio.

**Supplementary Table S4: Incidence rate ratios and differences for leukaemia comparing children prenatally exposed to nitrofurantoin and pivmecillinam. Restricted to children who are followed for the entire pregnancy.^a^**

|  |  | Any leukaemia | | | | |  | Lymphoid leukaemia | | | | AML |
| --- | --- | --- | --- | --- | --- | --- | --- | --- | --- | --- | --- | --- |
|  | n | cases | IRR (95% CI) | wIRR^b^ (95% CI) | IRD pr 100,000 person-years (95% CI) | wIRD^b^ pr 100,000 person-years (95% CI) | cases | IRR (95% CI) | wIRR^b^ (95% CI) | IRD pr 100,000 person-years (95% CI) | wIRD^b^ pr 100,000 person-years (95% CI) | cases |
| Pivmecillinam | 220 648 | 115 | reference |  |  |  | 95 | reference |  |  |  | 13 |
| Nitrofurantoin | 40 181 | 28 | 1.37 (0.89;2.11) | 1.32 (0.84;2.08) | 2.12 (-1.29;5.54) | 1.85 (-1.64;5.33) | 23 | 1.37 (0.87;2.16) | 1.29 (0.78;2.15) | 1.76 (-1.19;4.72) | 1.40 (-1.75;4.54) | <5 |
| *2 or more prescription fills* | | | | | | | | | | | | |
| Pivmecillinam | 44 403 | 21 | reference |  |  |  | 15 | reference |  |  |  | <5 |
| Nitrofurantoin | 5210 | 6 | 2.37 (0.96;5.87) | 1.75 (0.60;5.09) | 7.40 (-4.21;19.02) | 4.00 (-6.01;14.01) | <5 | - | - | - | - | <5 |
| *Trimester of exposure* | | | | | | | | | | | | |
| *1st trimester* | | | | | | | | | | | | |
| Pivmecillinam | 65 472 | 24 | reference |  |  |  | 22 | reference |  |  |  | <5 |
| Nitrofurantoin | 10 702 | 7 | 1.84 (0.79;4.27) | 2.01 (0.87;4.67) | 3.44 (-2.91;9.80) | 4.09 (-2.76;10.95) | 5 | 1.43 (0.54;3.78) | 1.57 (0.59;4.14) | 1.63 (-3.61;6.86) | 2.11 (-3.54;7.75) | <5 |
| *2nd trimester* | | | | | | | | | | | | |
| Pivmecillinam | 79 020 | 39 | reference |  |  |  | 36 | reference |  |  |  | 0 |
| Nitrofurantoin | 14 303 | 6 | 0.88 (0.37;2.07) | 0.63 (0.23;1.77) | -0.69 (-4.88;3.49) | -2.01 (-5.60;1.58) | 5 | 0.79 (0.31;2.01) | 0.69 (0.24;1.93) | -1.08 (-4.87;2.72) | -1.59 (-5.19;2.01) | <5 |
| *3rd trimester* | | | | | | | | | | | | |
| Pivmecillinam | 106 779 | 61 | reference |  |  |  | 45 | reference |  |  |  | 11 |
| Nitrofurantoin | 18 599 | 17 | 1.51 (0.85;2.67) | 1.48 (0.81;2.69) | 3.40 (-2.35;9.15) | 3.17 (-2.72;9.06) | 13 | 1.48 (0.76;2.87) | 1.38 (0.68;2.82) | 2.48 (-2.62;7.57) | 1.96 (-3.09;7.02) | <5 |

^a^Results from mixed effects Poisson models.

^b^Inverse probability of treatment weights including calendar year at birth, maternal age, parity, maternal history of cancer before pregnancy, prescription fills for immunosuppressants, systemic corticosteroids, and systemic antibiotics before start of pregnancy, maternal smoking status during first trimester, and child sex. In Finland, birth year was not balanced after weighting and added to the outcome model.

IRD: Incidence rate difference; IRR: Incidence rate ratio; wIRD: weighted incidence rate difference; wIRR: weighted incidence rate ratio.

**Supplementary Table S5: *Post hoc* analysis of incidence rate ratios and differences for leukaemia comparing children prenatally exposed and unexposed to nitrofurantoin.^a^**

|  |  | Any leukaemia | | | | |  | Lymphoid leukaemia | | | |
| --- | --- | --- | --- | --- | --- | --- | --- | --- | --- | --- | --- |
|  | n | cases | IRR (95% CI) | wIRR^b^ (95% CI) | IRD pr 100,000 person-years  (95% CI) | wIRD^b^ pr 100,000 person-years (95% CI) | cases | IRR (95% CI) | wIRR^b^ (95% CI) | IRD pr 100,000 person-years (95% CI) | wIRD^b^ pr 100,000 person-years (95% CI) |
| Unexposed | 2 254 684 | 1435 | reference |  |  |  | 1115 | reference |  |  |  |
| Nitrofurantoin | 44 091 | 32 | 1.23 (0.87;1.76) | 1.21 (0.72;2.03) | 1.44 (-1.25;4.13) | 1.43 (-2.80;5.65) | 26 | 1.28 (0.86;1.88) | 1.28 (0.71;2.29) | 1.29 (-1.12;3.70) | 1.45 (-2.42;5.32) |
| 2 or more prescription fills | | | | | | | | | | | |
| Unexposed | 2 254 684 | 1435 | reference |  |  |  | 1115 | reference |  |  |  |
| Nitrofurantoin | 5643 | 6 | 1.75 (0.79;3.91) | 1.73 (0.71;4.21) | 4.64 (-4.04;13.33) | 4.94 (-5.43;15.30) | <5 | - | - | - | - |
| Trimester of exposure | | | | | | | | | | | |
| 1st trimester | | | | | | | | | | | |
| Unexposed | 2 287 087 | 1460 | reference |  |  |  | 1136 | reference |  |  |  |
| Nitrofurantoin | 11 688 | 7 | 1.02 (0.48;2.14) | 0.91 (0.41;2.00) | 0.11 (-4.58;4.80) | -0.70 (-6.11;4.72) | 5 | 0.91 (0.38;2.21) | 0.81 (0.32;2.04) | -0.42 (-4.33;3.50) | -1.17 (-5.72;3.37) |
| 2nd trimester | | | | | | | | | | | |
| Unexposed | 2 283 118 | 1461 | reference |  |  |  | 1136 | reference |  |  |  |
| Nitrofurantoin | 15 657 | 6 | 0.65 (0.29;1.44) | 0.54 (0.23;1.26) | -2.20 (-5.42;1.02) | -3.71 (-7.43;0.01) | 5 | 0.68 (0.28;1.63) | 0.57 (0.23;1.45) | -1.58 (-4.48;1.32) | -2.73 (-6.13;0.68) |
| 3rd trimester | | | | | | | | | | | |
| Unexposed | 2 275 852 | 1445 | reference |  |  |  | 1124 | reference |  |  |  |
| Nitrofurantoin | 20 460 | 21 | 1.72 (1.12;2.66) | 1.78 (1.04;3.05) | 4.45 (-0.14;9.04) | 4.91 (-1.08;10.90) | 16 | 1.67 (1.01;2.74) | 1.56 (0.66;3.70) | 3.22 (-0.77;7.21) | 2.86 (-3.99;9.71) |

^a^Results from mixed effects Poisson models.

^b^Inverse probability of treatment weights including calendar year at birth, maternal age, parity, maternal history of cancer before pregnancy, prescription fills for immunosuppressants, systemic corticosteroids, and systemic antibiotics before start of pregnancy, maternal smoking status during first trimester, and child sex. In Finland, birth year was not balanced after weighting and added to the outcome model.

IRD: Incidence rate difference; IRR: Incidence rate ratio; wIRD: weighted incidence rate difference; wIRR: weighted incidence rate ratio.

**
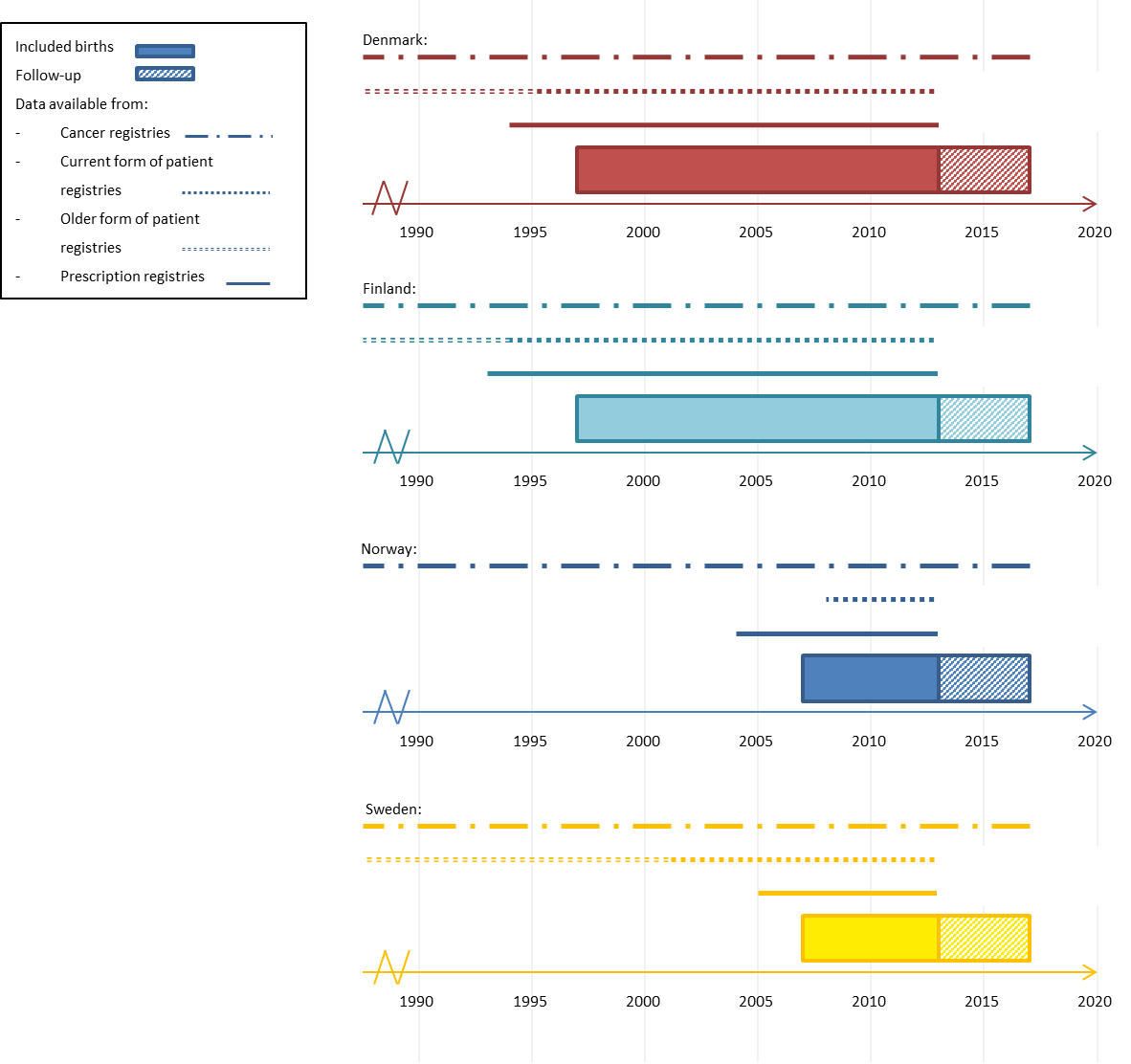
**

**Supplementary Figure 1:** Data included in the study


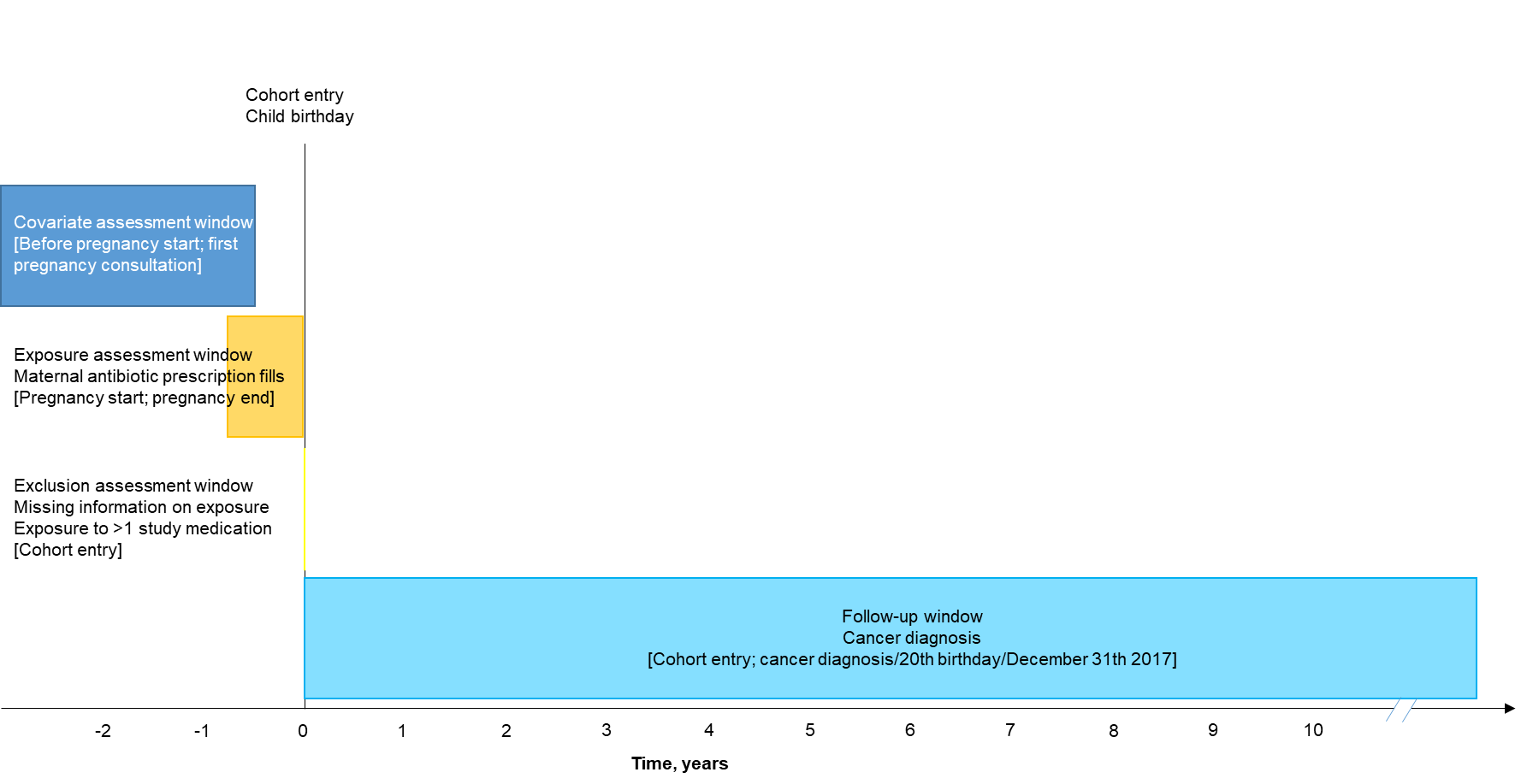


**Supplementary Figure** **2**: Study design diagram.


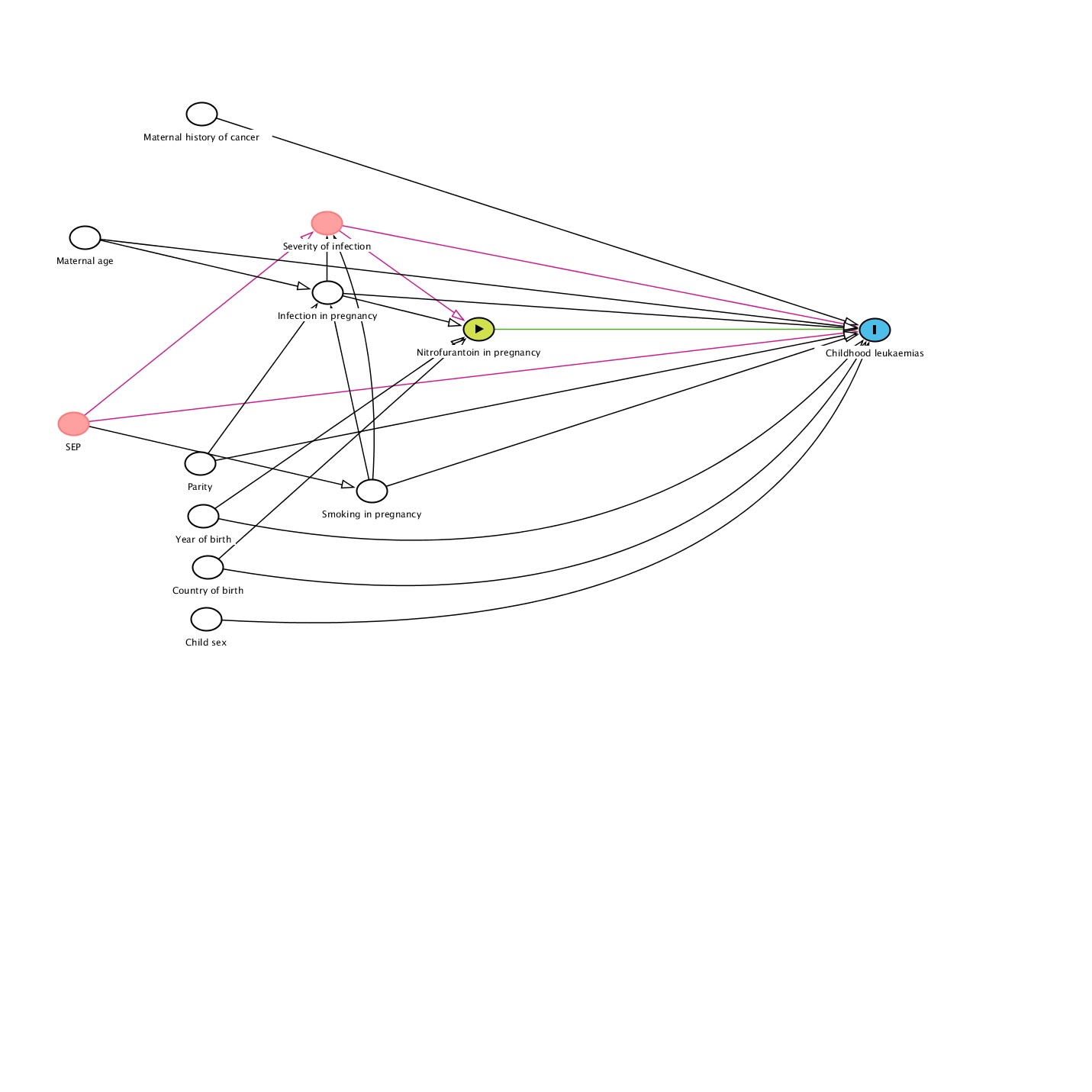


**Supplementary Figure 3**: Directed acyclic graph. SEP: socio-economic position.
